# Before and beyond trust: reliance in medical AI

**DOI:** 10.1136/medethics-2020-107095

**Published:** 2021-08-23

**Authors:** Charalampia (Xaroula) Kerasidou, Angeliki Kerasidou, Monika Buscher, Stephen Wilkinson

**Affiliations:** 1 Department of Sociology, Lancaster University, Lancaster, UK; 2 The Ethox Centre, Nuffield Department of Population Health, University of Oxford, Oxford, UK; 3 Department of Politics, Philosophy, & Religion, Lancaster University, Lancaster, UK

**Keywords:** ethics, information technology

## Abstract

Artificial intelligence (AI) is changing healthcare and the practice of medicine as data-driven science and machine-learning technologies, in particular, are contributing to a variety of medical and clinical tasks. Such advancements have also raised many questions, especially about public trust. As a response to these concerns there has been a concentrated effort from public bodies, policy-makers and technology companies leading the way in AI to address what is identified as a "public trust deficit". This paper argues that a focus on trust as the basis upon which a relationship between this new technology and the public is built is, at best, ineffective, at worst, inappropriate or even dangerous, as it diverts attention from what is actually needed to actively warrant trust. Instead of agonising about how to facilitate trust, a type of relationship which can leave those trusting vulnerable and exposed, we argue that efforts should be focused on the difficult and dynamic process of ensuring reliance underwritten by strong legal and regulatory frameworks. From there, trust could emerge but not merely as a means to an end. Instead, as something to work in practice towards; that is, the deserved result of an ongoing ethical relationship where there is the appropriate, enforceable and reliable regulatory infrastructure in place for problems, challenges and power asymmetries to be continuously accounted for and appropriately redressed.

## Introduction

Artificial intelligence (AI) is changing healthcare and the practice of medicine as data-driven science and machine-learning technologies, in particular, are contributing to a variety of medical and clinical tasks.^1 2^ This is indicative of a broader structural shift in healthcare, as the increased digitisation of the sector is creating a complex and potentially lucrative clinical data ecosystem enabled by a new constellation of actors; namely global consumer technology corporations which now join the medical professionals, healthcare providers, pharmaceutical companies, manufacturers and regulators as another key player in the healthcare domain.^3^ In this landscape, new clinical-corporate alliances are being formed as clinicians come under pressure to use valuable resources such as clinical data for better, cheaper, more efficient health services, while the corporations are seeking opportunities to establish themselves in (and arguably profitably mine) this growing market.^4^

However, such alliances also raise concerns as controversial data initiatives and scandals continue to hit the headlines.^5–7^ In policy circles, in particular, these concerns have attracted a focus on trust, with many hoping that fostering public trust would dispel them and make it easier for AI technology^1^ to be accepted.^8–10^ Governments, advocacy groups and other national and international organisations are putting together guidelines and codes of ethics for AI governance in an effort to engender public confidence.^2^ For example, the European Commission places trust at the heart of its framework for Trustworthy AI, seeking to foster confidence in the technology’s development and applications by identifying trust as ‘the bedrock of societies, communities, economies and sustainable development’.^11^ The UK’s National Health Service (NHS) has developed a code of conduct for AI that articulates the ethical principles that should guide data-driven care.^12^ The tech industry has also been an active participant in these attempts to foster trust, setting up ethics advisory boards and developing its own codes of conduct, in order to show that it takes ethics seriously and bolster AI’s trustworthiness.^3^

Efforts to develop ethical principles and governance of AI have arguably foregrounded ethics as an important way of addressing the issue of public trust.^14 15^ However, their effectiveness to engender trust is questionable, while their largely voluntary nature ignores the reasons why governance is needed in the first place. In this paper, we challenge this focus on trust. Drawing broadly from philosophy, we understand trust as a type of relation that cannot merely be required, prescribed or coaxed.^4^ It should be freely given and, in putting one’s trust in another, one makes oneself vulnerable and dependent on the goodwill of the trustee. Trust occurs when one feels reasons to trust.^16–18^ As things stand, the public has little evidence that reasons to trust these new actors exist and that their long-voiced concerns are taken seriously. As such, we argue that a focus on trust as the basis on which a relationship between this new technology and the public is built is, at best, ineffective, at worst, inappropriate as it diverts attention from what is needed to actively warrant trust. By fixating on "improving" or "maintaining" trust in AI, policy-makers and technology developers are failing to provide reasons to trust and risk leaving the public vulnerable to Big Tech companies that are entering the healthcare space without evidence of their trustworthiness and commitment to the public good,^19^ and a clear course of holding them accountable if and when things go wrong.^20^ Instead, we argue that efforts should be focused on the difficult and dynamic process of ensuring reliance. Although in everyday language, trust and reliance are sometimes used interchangeably, there is a clear normative distinction between the two. Whereas trust normally denotes a relationship underpinned by the trustee’s goodwill towards the trustor, reliance is about ensuring predictability of behaviour.^17^ In the context of AI in healthcare, reliance can be underwritten by strong legal and regulatory frameworks that protect the public and ensure fair collaborations that serve the public good. From there, trust could emerge but not merely as a means to an end. Instead, as something to work in practice towards; that is, the deserved result of an ongoing ethical relationship where there is the appropriate, enforceable and reliable regulatory infrastructure in place for problems, challenges and power asymmetries to be continuously accounted for and appropriately redressed.

## Public (dis)trust

It is often stated as a fact that there is a crisis of public trust in AI which risks putting in danger the promise of AI in healthcare by ‘stifling innovation’ and resulting in unnecessary 'opportunity costs’.^22^ For example, in 2018, the House of Lords report on AI in the UK stressed that ‘Maintaining public trust over the safe and secure use of their data is paramount to the successful widespread deployment of AI and there is no better exemplar of this than personal health data’.^9^ In 2019, UK’s Secretary of State, Matt Hancock, and the Minister for Innovation, Baroness Blackwood, highlighted trust—underlined by ‘ethics, transparency and the founding values of the NHS’—as the key to UK’s healthcare AI policy success.^12^ And writing for *The Lancet* in 2019, Morley, Taddeo and Floridi identify a ‘deficit of trust’ as the ‘fundamental problem’ to ‘unlocking’ the opportunities that collaborations such as that between Google Health and the NHS can achieve.^23^

To date, few studies have been conducted to gauge the views of the public on the use of AI in sectors such as healthcare. Those that exist reveal a complex picture with responses that are thoughtful, pragmatic and largely positive. For example, a 2017 survey conducted on behalf of The Royal Society found that its participants were ‘highly positive’ about machine learning’s potential in the health sector.^24^ Also, a key finding of a 2018 report prepared for the Academy of Medical Sciences which asked members of the public, patients and healthcare professionals about their views on future data-driven technologies was that ‘There is optimism about new technology in healthcare. Participants felt new technologies in general could increase efficiency, improve success rates of diagnoses and save administrative and diagnostic time meaning clinicians could spend more time on patient care.’^25^

Besides this optimism regarding the potential benefits of AI, these surveys also foreground questions and concerns on the opaque relationship that is being developed between public–private stakeholders, the danger of profiteering and of commercial interests clashing with the ethical values of healthcare and the need for appropriate regulation to carefully govern these new partnerships. These concerns, and associated calls for more regulation, are not new.^26–28^ For example, in 2013 when the now infamous case of care.data—an English initiative designed to allow the repurposing of primary care medical data for research and other purposes—occurred, issues such as the increasing commercialisation of healthcare, doubts over the new commercial partners’ commitment to the public good, and concerns over the loss of privacy when private actors enter the landscape, were also raised by the public.^29 30^ The project ended up being rejected and withdrawn.^5^

Since then, care.data is often cited as a cautionary tale about the importance of public trust and the costly danger of losing it.^32 33^ However, this is only half of the story. As subsequent studies have shown, the other half is that people’s views were not ‘taken seriously’.^30^ Public trust cannot be coaxed with narrowly focused public relations exercises that merely seek to ‘capture’ the public; namely convince them of the legitimacy of decisions already taken *for* them rather than *with* them.^29^ Sociological research has demonstrated that the public’s relationship with science and technology is too complex to be characterised by a simple trust/distrust relationship,^34^ and the aforementioned studies confirm that. Past research has shown that science policy strategies that insist on addressing the “crisis of public trust” by trying to improve trust using top-down strategies such as informing/educating/communicating but without seriously engaging with and addressing the institutional reasons that led to public distrust, are condemned to repeat the same mistakes.^35^ Echoing Banner’s words, public trust is often cited as a cornerstone of better data use in the NHS and beyond, yet unless we address the conditions necessary for creating an environment worthy of trust in this new clinical-data ecosystem, it will remain elusive.^36^

## Trust and ethical codes

Fears of a deficit of public trust along with the host of ethical concerns that these new technologies introduce have triggered a surge of investment into ethical AI by governments, tech companies and other national and international organisations.^37^ This proliferation of principles, codes of ethics and practice, and PR campaigns has arguably foregrounded ethics and its importance.^14 15^ However, they are not without criticism. Many caution against their limitations, such as the difficulty of translating abstract ethical principles into the practical guidance needed by designers to address particular use cases and applications.^38 39^ Furthermore, reports show that the effectiveness of voluntary codes and guidelines is minimal as they fail to change the practices of tech professionals.^40 41^ As O’Neil argues, ethics governance practices and principles, such as confidentiality or consent do not confirm trust, but rather presuppose it.^42^ On this view, unless the companies who develop AI technologies are already seen as trustworthy, codes of conduct and ethical guidelines will not provide sufficient reasons to trust. Floridi also notes that ethics as a form of regulation, including self-regulation, ‘can only function in an environment of public trust and clear responsibilities more broadly’.^43^ The implication for public trust is that ethical principles alone, without a clear relationship and a strong legal framework, cannot provide enough motivation for trust. So, trying to address this perceived trust deficit through the introduction of ethics rules and self-regulations is ineffective as it puts "the cart before the horse".

Some also argue that codes of conduct prescribe ethics in a narrow and formalised way, where concerns raised by the public fall outside an agenda already set by policy-makers and traditional medical ethics.^15^ Even more so, the largely voluntary nature of ethical codes of conduct ignores the reasons why they are needed in the first place. The strong oversight and accountability mechanisms that could evidence genuine ethical commitments and concretely address the public’s concerns are not there. Furthermore, reports on Big Tech’s powerful lobbying, and its monetary influence on the ethics debate^44–46^ lend further credibility to criticisms of "ethics-washing". This is a phenomenon that is strategically used (and abused), first, to lend credibility and signal the moral standing of a company within a landscape where ethics is deemed to be the ‘hottest product in Silicon Valley’s hype-cycle’,^47^ and second, to divert attention from legal and regulatory forms of governance.^48–50^ While the former provides further reasons to question the trustworthiness of these new actors, the latter poses a particular problem for the healthcare sector which is traditionally governed by strict professional codes on safety and accountability, as it raises the question; what might we miss when attention is diverted from legal rules and regulations?

Even though these technologies are entering the healthcare sector, it is questionable whether we are ready to use them safely and ethically. In the UK, a country which seeks to become a world leader in health^9^, a report which assessed the state of AI in the sector concluded that the NHS IT infrastructure is not fit for AI yet.^51^ In 2019, Eric Topol, who led the government-commissioned Topol Review in the UK,^52^ warned that the state of AI hype has far exceeded the state of AI science,^53^ and in 2020 he called for updated regulations, standards and pathways of transparency that will require, not just retrospective studies as is currently the case, but actual clinical trials to prove the safety of AI medical tools.^54^ The oft-claimed superiority of AI’s diagnostic performance over that of doctors does not always hold when put under careful scrutiny.^55^ And others warn that we still lack a clear regulatory pathway for AI medical devices,^56^ and the necessary evaluations to check whether a new AI system in practice does more good than harm^57^ resulting in allowing unsafe technologies ‘into the wild’.^58^ In Gould’s words, the CEO of NHSX, who after meeting in 2020 with the UK’s relevant AI regulators identified ‘gaps, lots of regulators on the pitch, and a lack of clarity on both standards and roles’, ‘We aren’t there yet’.^59^

## Before and beyond trust

Trust has been theorised across many disciplines without a consensus on its definition.^6^ Drawing broadly from philosophy we could say that trust relationships take the form of: A trusts B to x. A trusts B to perform a specific action x, when A, the trustor, believes that B, the trustee, possesses the appropriate knowledge and skills to perform the entrusted action, and also goodwill towards A.^16–18^ It is given only when people feel they have reasons to trust. According to Baier: ‘Trust me!’ is for most of us an invitation which we cannot accept at will—either we do already trust the one who says it, in which case it serves at best as reassurance, or is responded to with, ‘Why should and how can I, until I have cause to?’^16^ However, this is exactly what the public is asked to do with these corporate actors even after they have, times and again, expressed their reasons for not trusting. Furthermore, as the case of care.data demonstrates, asking people to trust, when they question whether they have good reasons to do so, can be counterproductive. This is because, given the opportunity, people will retreat from a situation that could make them dependent on or vulnerable to someone they consider of questionable trustworthiness.^60^

This brings us to another basic characteristic of trust. In trust relationships, the trustor can become vulnerable to the trustee, and dependent on their goodwill.^16^ Vulnerability and the power imbalance it entails are at the heart of healthcare.^62^ As such, it is governed by strict professional codes and strong ethical commitments on safety, and clear, enforceable pathways to accountability. However, as Mittelstadt explains, while AI borrows from medical ethics for developing its own ethical frameworks, it lacks the ‘(1) common aims and fiduciary duties, (2) professional history and norms, (3) proven methods to translate principles into practice and (4) robust legal and professional accountability mechanisms' of medicine.^63^ These existing legal, regulatory and ethical gaps mean that it is not yet clear what happens if and when things go wrong with AI in healthcare. For example, legal scholars demonstrate that due to the complexity and gaps in the existing English law that addresses liability in the use of AI for clinical decision making, the tech companies seem to be protected while clinicians become morally and legally answerable for the potential defects of the AI system they choose to use.^20^ Also, the recent controversy over Babylon Health’s ‘chatbot’ triage service^7^ brings into question the openness and readiness of tech companies to address legitimate concerns about the safety of their AI health products,^62^ and highlights the legal and regulatory gap that exists over their use.^64^ By asking the public to trust AI, and as such the tech companies driving this innovation, what is asked of them is to accept the risk that these companies are free to decide whether they will confirm or betray public trust. But how could the public reasonably take such a position, when they feel that they don’t yet have reasons to trust? It seems inappropriate to ask the public to accept that position of vulnerability. In this light, trust seems to be an inappropriate, if not a dangerous, basis on which to base our relationship to AI.

There is, however, another way to approach this relationship that could avoid the pitfalls of trust while addressing the public’s concerns. We argue for reliance. Reliance can be understood as ‘dependence based on the likely prediction of another’s behaviour’,^17^ and while some understand trust as comprising reliance,^8^ there is a clear normative distinction between the two. Whereas trust, and the related trustworthiness, denote a moral characteristic or attitude, reliance and reliability are about predictability of behaviour without any reference to moral commitments or values.^18^ A relationship of reliance is based on reasonable expectations, proven capacity, open communication and clear and enforceable systems of responsibility and accountability.^66^ When a relationship of reliance breaks down, blame is sought externally.^67^ This is why, in contrast to trust where feelings of betrayal might be evoked, relationships of reliance necessitate clear pathways of responsibility and accountability. What ensures reliance is the presence of self-interest that secures the partner’s commitment to the relationship, including the desire to avoid loss or penalty.^66^

Of course, this is not to say that things cannot go wrong in relationships of reliance. The risk is always there considering that we rely on someone when it is necessary to do so.^69 70^ However, in contrast to trust, there is no emotionally invested acceptance of this risk. Instead, mechanisms such as formal rules, contracts, regulations and systems of accountability, devised, implemented and overseen by independent and accountable governments and supranational organisations, are expected to offset it, hence protecting the public good while offering reasonable and equitable benefits to all parties. A mandatory, coherent and enforceable legal and regulatory framework would redress the power asymmetries between partners, ensure predictability of behaviour and accountability, and help establish a successful relationship based on openness, competence and reliability.

## Conclusion

This paper argues for a shift in AI debates from trust to reliance, making the case that we should not be distracted by a quest on how to trust AI technologies when we don’t know if we can rely on them yet. As Sheehan *et al* remind us, this is not a negative conclusion, but one that recognises the conflict and power imbalance between healthcare and commercial interests^71^ and, importantly, acknowledges the fact that such imbalances affect the rigour with which these technologies can be evaluated, regulated and introduced. Advocating for and insisting on appropriate and enforceable regulation neither ends the discussion nor closes down the ethical debate. As the case of care.data illustrates,^29–31^ what constitutes appropriate and acceptable regulation is not straightforward. So, how do we judge these new and evolving technologies to be sufficiently safe or not? How do we ensure continuous monitoring as these machine-learning algorithms adjust, train and learn, or when they are applied in practice? How do we ensure oversight but also factor in uncertainty and risk? How do we judge that there aren’t other, simpler, more transparent, and more robust solutions for the task at hand? It is important that these questions, and many like them,^53 72^ are debated and decided not just in healthcare but across the AI sector. From there, trust could emerge but not as a means to an end. Instead, as something to work in practice towards; that is, the deserved result of an ongoing ethical relationship where there is the appropriate, enforceable and reliable regulatory infrastructure in place for problems, challenges and power asymmetries to be continuously accounted for and appropriately redressed. While important work has already started,^73^ there is still much to be done. Shifting our attention from trust to reliance will refocus the debate and allow us the space and time to carefully and publicly consider these urgent matters.

## Data Availability

Data sharing not applicable as no datasets generated and/or analysed for this study.

## Footnotes

We understand AI not as a stand-alone tool, but as a socio-technical construct which brings together the social, technical, regulatory, ethical, political, imaginary.^13^

The organisation *Algorithm Watch* is in the process of compiling an AI Ethics Guidelines Global Inventory which currently counts more than 90 such sets https://inventory.algorithmwatch.org/ [Accessed 29 Apr 2021]

For example, Microsoft and Google have developed principles for ethical AI, and along with Amazon and Facebook were some of the founding members of the *Partnership on AI to benefit People and Society*. In 2016, DeepMind Health appointed an Independent Review Panel to scrutinise their work with the NHS. The panel was later disbanded after the company’s controversial take over by Google Health.

One can talk about trust between individuals -personal trust-, or between groups or collectives such as companies and institutions, -institutional, apersonal or impersonal trust. As Kerasidou argues, in so far as a trust relationship is between two moral actors, being individuals or collectives/institutions, the type of relational trust we describe here applies.^21^

Interestingly, similar initiatives launched in Scotland and Wales were less controversial and more successful. See McCartney’s account of why and how the devolved nations did things differently.^31^

For a comprehensive account on the literature on trust particularly aimed towards the healthcare sector, see^61^

Babylon’s chatbot is a symptom-checker app, already used by some NHS Trusts, which identifies possible causes or gives advice to the user such as “go to the hospital”. When David Watkins, a consultant oncologist at the Royal Marsden NHS Foundation Trust who had repeatedly questioned the app’s safety, went public with his concerns in February 2020, Babylon Health described him as a ‘troll’.^65^

Trust is typically understood as a relational concept between two entities that comprises two elements; reliance plus something. Much of the literature focuses on this second clause; i.e., Baier talks about goodwill,^16^ Holton about the ‘participant stance’^68^ while Hawley about a standing commitment of the trustee towards the trustor.^18^ Thomson presents a different account of reliance not as a constitutive feature of trust.^69^
